# Culture surface protein coatings affect the barrier properties and calcium signalling of hESC-RPE

**DOI:** 10.1038/s41598-020-79638-8

**Published:** 2021-01-13

**Authors:** Taina Viheriälä, Juhana Sorvari, Teemu O. Ihalainen, Anni Mörö, Pyry Grönroos, Sabrina Schlie-Wolter, Boris Chichkov, Heli Skottman, Soile Nymark, Tanja Ilmarinen

**Affiliations:** 1 Faculty of Medicine and Health Technology, BioMediTech, Tampere University, Tampere, Finland; 2 Institute for Multiphase Processes, Leibniz University of Hannover, Hannover, Germany; 3 Institute of Quantum Optics, Leibniz University of Hannover, Hannover, Germany

**Keywords:** Cell biology, Stem cells, Diseases, Molecular medicine

## Abstract

Human pluripotent stem cell-derived retinal pigment epithelium (RPE) transplantation is currently under evaluation as treatment for macular degeneration. For therapeutic applications, cryostorage during cell production is typically needed with potential consequences to cell functionality. We have previously shown that the culture substrate affects human embryonic stem cell-derived RPE (hESC-RPE) properties in fresh cultures. Here, we aimed to further identify the role of RPE basement membrane proteins type IV collagen (Col-IV), laminin (LN), and nidogen-1 in the maturation and functionality of hESC-RPE after cryopreservation. In addition to cell attachment and morphology, transepithelial electrical resistance, expression of key RPE proteins, phagocytosis capacity and Ca^2+^ signalling were analysed. After cryostorage, attachment of hESC-RPE on culture surfaces coated with Col-IV alone was poor. Combining Col-IV and LN with or without nidogen-1 significantly improved cell attachment and barrier properties of the epithelium. Furthermore, functional homogeneity of the hESC-RPE monolayer was enhanced in the presence of nidogen-1. Our results suggest that the choice of coating proteins for the cell culture may have implications to the functional properties of these cells after cryostorage cell banking.

## Introduction

Retinal pigment epithelium (RPE) cells form a highly polarised pigmented monolayer between the neural retina and the choriocapillaris at the back of the eye. RPE plays a central role in the maintenance of a healthy retina. Photoreceptor function and survival is supported by RPE via multiple processes responsible for the transport of nutrients, waste products, ions and fluid between the choroidal blood supply and the subretinal space as well as photoreceptor outer segment (POS) phagocytosis and visual pigment regeneration^1^. Dysfunction of RPE cells leads to the death of photoreceptors, resulting in progressive retinal degenerative diseases, such as age-related macular degeneration (AMD), a major cause of blindness among the elderly in the developed countries^2^. Treatment options for these diseases are currently limited and mostly only delay disease progression. However, replacement of the damaged RPE with human pluripotent stem cell (hPSC)-derived healthy RPE (hPSC-RPE) has been considered as a promising treatment strategy^3^.

For clinical applications, the hPSC-RPE cells need to be well-characterized, functionally competent and consistent in quality^4,5^. Cryopreservation of cells is an important part of clinical production process, enabling increased shelf-life and storage of large, quality-controlled, batches of cells^4,5^. However, cryopreservation may affect cellular properties such as adhesion to extracellular matrix (ECM) and following downstream signalling, which is vitally important for appropriate cell physiology and functionality. For example, it has been shown that cryopreservation decreases the expression of certain adhesion molecules on CD34 + hematopoietic progenitor cells^6^. Human umbilical vein endothelial cells have also been shown to be vulnerable to cryopreservation which can lead to lowered angiogenic functionality^7^. In the eye, RPE sits on an ECM called Bruch’s membrane which provides structural support and cues affecting e.g. cell differentiation, morphology, and function. The upper part of the Bruch’s membrane forms the RPE basement membrane, which is rich in collagen type IV (Col-IV), laminin (LN, types LN111, LN332, LN511, and LN521), nidogen-1 (Nid-1), fibronectin, hyaluronic acid, heparan sulfate, and chondroitin/dermatan sulfate^8–10^. RPE cells express various integrin subunits (i.e. α1-6, and β1), and proteoglycans (i.e. perlacan), forming receptors for ECM proteins such as laminins, collagens, and Nid-1^10–14^. RPE cells can be differentiated and grown in vitro to form confluent monolayers on a variety of ECM mimicking culture surface coatings, such as mixed ECM substrates including Matrigel, purified/recombinant ECM proteins like Col-IV and LN, or synthetic substrates such as Synthemax II-SC^15–19^. Nevertheless, we and others have previously shown that the choice of protein composition for coating cell culture surfaces has major effects on RPE differentiation efficiency, as well as RPE structure, basal lamina production and barrier properties of hPSC-RPE in fresh cultures^16,20^. However, comparative studies on the functional consequences of different protein coatings after cryopreservation are lacking.

RPE is part of the outer blood-retinal barrier which regulates the composition of the subretinal space enabling proper photoreceptor function. Tight junctions are an important component of tissue barriers and their permeability and selectivity is regulated by claudins, especially claudin-19 in human RPE^21^. The barrier properties of RPE have been extensively studied, and it has been shown that endothelial cell secreted factors regulate RPE basement membrane assembly, launching integrin-mediated Rho GTPase signalling that modulate RPE tight junctions and enhance RPE barrier function^22,23^. However, the effects of the cell culture surface protein composition on the maturation of hPSC-RPE tight junctions during differentiation have not been reported before. Another essential aspect for RPE physiology is calcium (Ca^2+^) signalling, as several critical RPE functions rely on Ca^2+^-dependent regulatory mechanisms^24^. Light-induced increase of adenosine triphosphate (ATP) in the subretinal space is an important activator of Ca^2+^ signalling in RPE, affecting e.g. RPE transport processes that are involved in the regulation of subretinal space hydration and chemical composition, and influence retinal adhesion^25,26^. Consequently, faults in Ca^2+^ signalling in the transplanted hPSC-RPE cells could contribute to unsuccessful cell therapy. Yet, only a few studies on Ca^2+^ signalling of hPSC-RPE exist^5,27–29^. We have previously shown, that functional voltage-gated Ca^2+^ channels are present in human embryonic stem cell (hESC) –derived RPE^30^. However, the effects of different ECM protein coatings on hPSC-RPE Ca^2+^ signalling have not been previously studied.

To address the potential importance of ECM coatings in the orchestration of cellular functions and responses of hESC-RPE, we investigated the role of the key RPE basement membrane proteins Col-IV, LN, and Nid-1, to the in vitro maturation and functionality of the hESC-RPE after cryopreservation, focusing on barrier properties, phagocytosis and Ca^2+^ signalling. Here, we show that the composition of protein coating affects cell attachment after cryostorage and can lead to profound functional changes during hESC-RPE maturation. Our results also highlight the importance of examining the Ca^2+^ signalling properties, as previously suggested by others^5^, when evaluating the quality of hPSC-RPE.

## Results

### Col-IV alone does not support the formation of an intact hESC-RPE monolayer on culture inserts after cryopreservation

**Figure 1:**
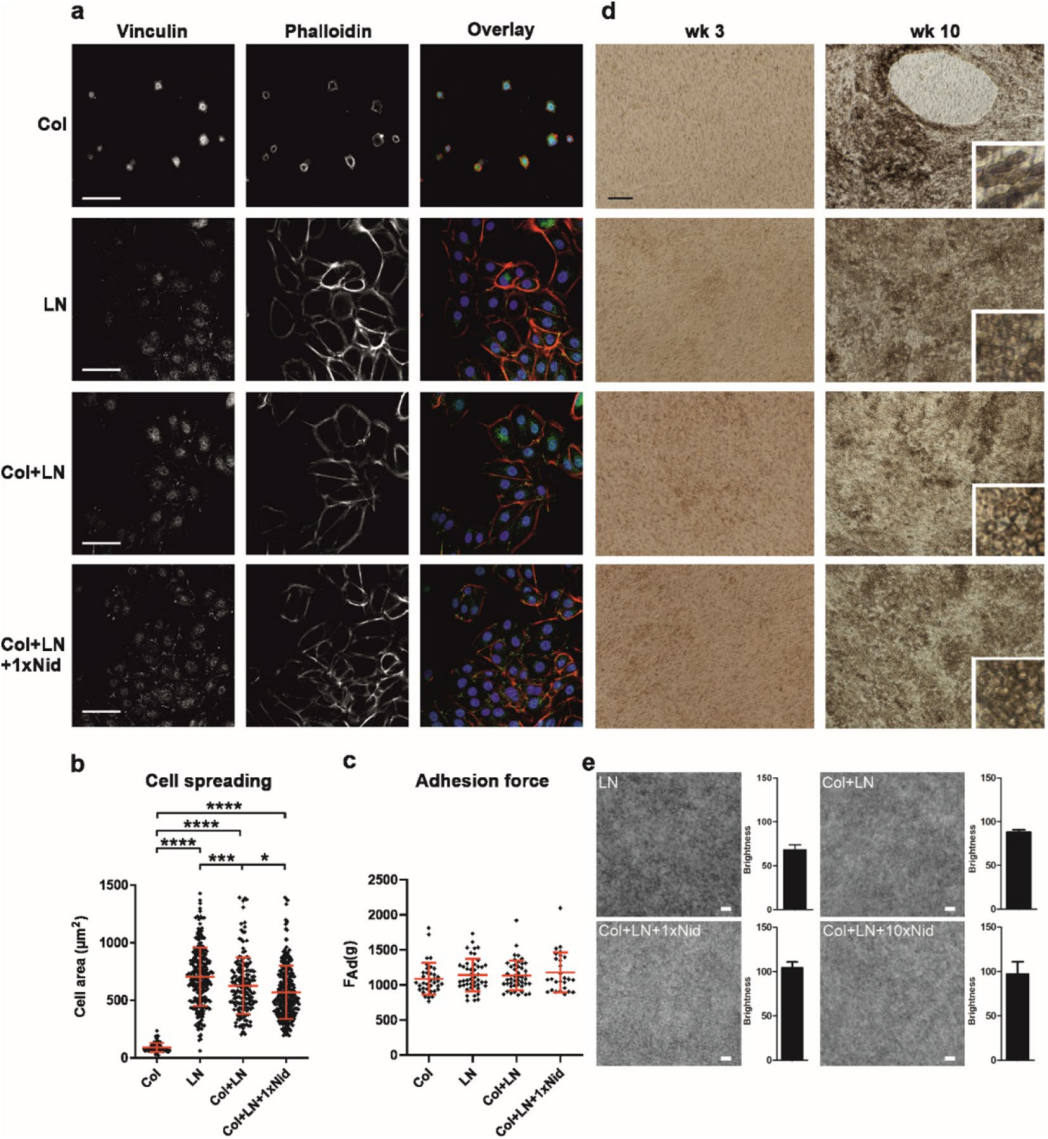
Adhesion of hESC-RPE on different protein coatings (all data shown for hESC-08/017). (**a**) Confocal single plane images showing the subcellular distribution of vinculin and filamentous actin 24 h after seeding. In the overlay, vinculin is shown in green, actin filaments are stained by phalloidin (red) and nucleus by DAPI (blue). Scale bar 50 µm. (**b**) Quantification of the cell area 24 h after seeding was done by outlining the periphery of cells based on F-Actin staining using ImageJ software. The analysed cell numbers were 80 (Col), 241 (LN), 168 (Col + LN) and 265 (Col + LN + Nid) and the results are given as mean ± SD. (**c**) Adhesion force (F_ad_) correlates with the force (g) needed to detach half of the adherent cells after centrifugation, values are given as mean ± SD. Measurements were done 24 h after seeding from 3 biological and 2–3 technical replicates for each coating. (**d**) Phase contrast micrographs of RPE cell morphology on different coatings 3 and 10 weeks after seeding. *p < 0.05, ***p < 0.001, ****p < 0.0001. (**e**) Pigmentation analysis of hESC-RPE after 8 weeks of seeding. Each image and bar represent the average intensity of five differential interference contrast (DIC) images (LSM800, Carl Zeiss, air immersion objective 20x). Brightness from each image was calculated using ImageJ and represents as mean ± SD. Difference in brightness was statistically significant between LN and all other coatings; LN + Col (p = 0.0079), LN + Col + 1xNid (p = 0.0079) and LN + Col + 10xNid (p = 0.0079). In addition, difference in brightness of LN + Col was statistically significant compared to LN + Col + 1xNid (p = 0.0079). Scale bar 20 µm. All statistics were performed with Mann–Whitney U. Col—collagen IV, LN—laminin, Nid—nidogen-1.

For successful cell culture, an environment allowing appropriate cellular responses is critical. In addition to chemical signals, interactions at the cell-material interface affect cellular signalling through cell-ECM adhesions^31^. Thus, we examined the adhesion and morphology of cryopreserved hESC-RPE (Table 1) 24 h after thawing and seeding on coverslips dip-coated with Col-IV, LN, Col-IV + LN, or Col-IV + LN + Nid-1. Cell flattening/spreading, formation of actin stress fibres providing force for cell adhesion and focal adhesion maturation on different coatings was studied by labelling the hESC-RPE for filamentous actin and important focal adhesion linker protein vinculin. On all other coatings except on Col-IV, hESC-RPE were well-spread, formed organised actin stress fibres, and showed recruitment of vinculin to focal adhesions and adherens junctions (Fig. 1a,b). On Col-IV, punctate vinculin staining was observed, but the cells exhibited rare cell–cell contacts, poor stress fibre formation and weak cell spreading (Fig. 1a,b). The strength of cell adhesion was examined using centrifugation force. Despite the low cell density and poor spreading on Col-IV, no differences in adhesion force between coatings were observed (Fig. 1c). Over the following 10-week culture time, an intact hESC-RPE monolayer on Col-IV alone was never reached and the cells had a fusiform morphology, unlike on all other coatings where hESC-RPE formed an intact pigmented epithelium with cobblestone cell morphology (Fig. 1d). The results indicate that in our culture conditions, LN either alone or in combination with Col-IV + /-Nid-1 is superior to Col-IV alone in supporting hESC-RPE cell attachment and formation of an intact epithelium. LN coating condition also produced the highest level of pigmentation in hESC-RPE, although the differences between LN alone or in combination with Col-IV + /-Nid-1 were modest (Fig. 1e).

**Table 1:** Number of replicates for all experiments.

	Cell lines	Coating	Biological n	Replicates/coating (n)						
				Technical replicates (total)						
				24 h	Wk 3	Wk 4	Wk 6	Wk 7–8	Wk 9–10	Wk 13
Cell adhesion force	hESC-08/017	Col	3	6–9 [m]						
		LN	3	6–9 [m]						
		Col + LN	3	6–9 [m]						
		Col + LN + 1xNid	3	6–9 [m]						
Cell area	hESC-08/017	Col	1	80 [c]						
		LN	1	168 [c]						
		Col + LN	1	241 [c]						
		Col + LN + 1xNid	1	266 [c]						
Morphology	hESC-08/017, hESC-11/013, hESC-13/012	All coatings	n/a		n/a				n/a	
TER	hESC-08/017, hESC-11/013, hESC-13/012	Col	6		12 [m]	32 [m]	34 [m]	46 [m]	56 [m]	
		LN	3		12 [m]	18 [m]	20 [m]	12 [m]	28 [m]	
		Col + LN	6		24 [m]	44 [m]	44 [m]	46 [m]	52 [m]	
		Col + LN + 1xNid	4		24 [m]	38 [m]	36 [m]	48 [m]	36 [m]	
IF	hESC-08/017, hESC-11/013	LN	2					2 [s]		
		Col + LN	2					2 [s]		
		Col + LN + 1xNid	2					2 [s]		
Phagocytosis assay	hESC-08/017	LN	1							5 [i] 519 [c]
		Col + LN	1							5 [i] 540 [c]
		Col + LN + 1xNid	1							5 [i] 528 [c]
		Col + LN + 10xNid	1							5 [i] 510 [c]
Ca-imaging	hESC-08/017 hESC-11/013	LN	2						465 [c]	555 [c]
		Col + LN	2						473 [c]	545 [c]
		Col + LN + 1xNid	2						591 [c]	643 [c]
		Col + LN + 10xNid	2						551 [c]	531 [c]
Pigmentation assay	hESC-08/017	LN	1					5 [i]		
		Col + LN	1					5 [i]		
		Col + LN + 1xNid	1					5 [i]		
		Col + LN + 10xNid	1					5 [i]		

[m] measurements, [c] cells, [s] stainings, [i] images, Col—collagen IV, LN—laminin, Nid—nidogen-1.

### Culture substrate coating affects the barrier formation of hESC-RPE in culture

**Figure 2:**
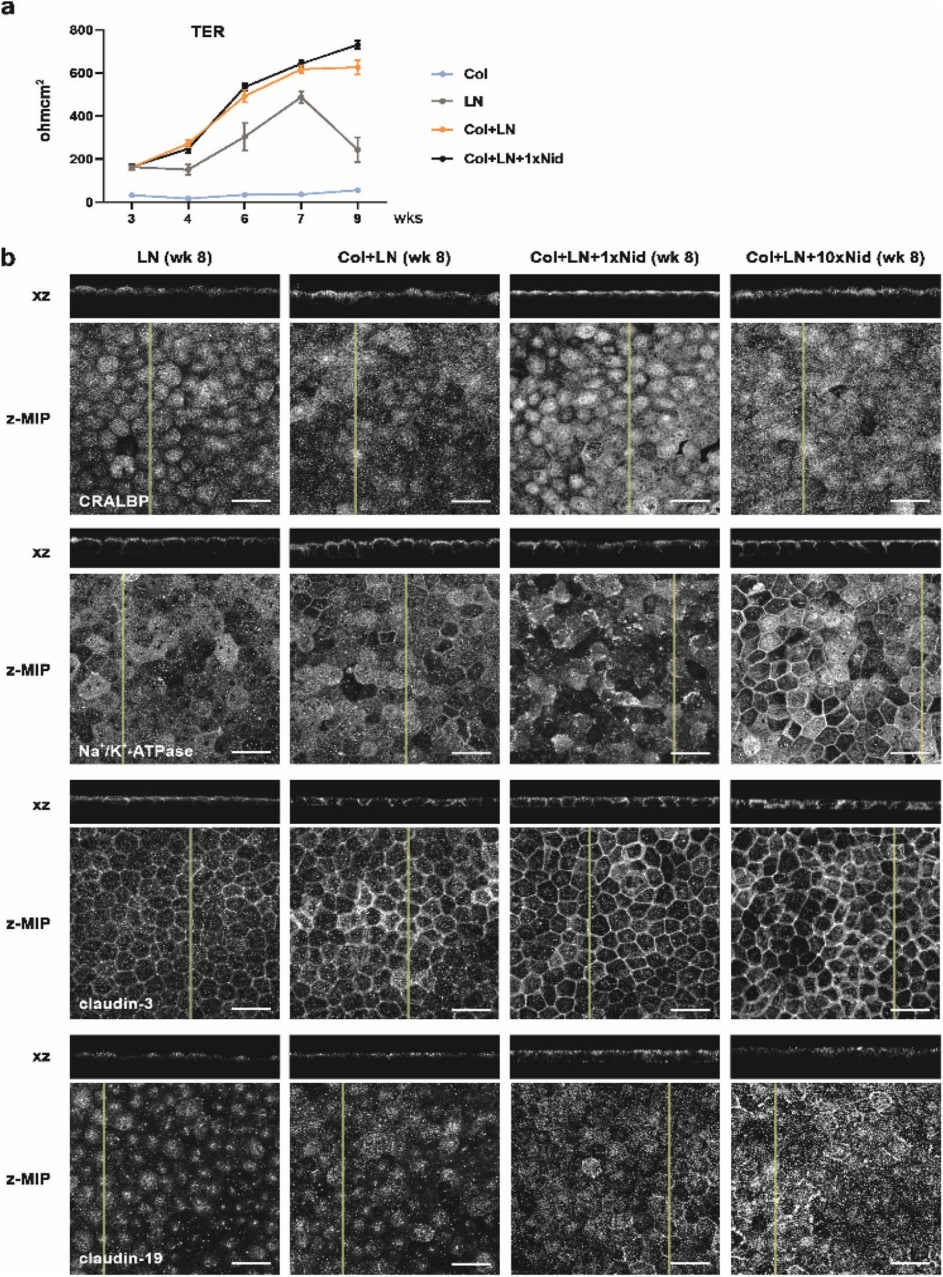
Barrier properties of hESC-RPE on different coatings. (**a**) TER of hESC-RPE on different coatings was measured over time. Data represents means ± SEM at 5 time points of 3 hESC-RPE lines from 3–6 biological and 6–28 technical replicates (Table 1). The differences in TER on LN compared to Col + LN are statistically significant at all time points (4 weeks p = 0.001, 6 weeks p = 0.035, 7 weeks p = 0.001, 9 weeks p < 0.0001). At 9-week time point the difference in TER between hESC-RPE on Col + LN + Nid and on Col + LN was statistically significant (p = 0.014). Statistical analysis was performed with Mann–Whitney U. (**b**) Representative (shown for hESC-08/017, Table 1) laser scanning confocal microscopy z-maximum intensity projections (z-MIP) and yz cross-sections (MIP from 10 sections) showing expression and subcellular localisation of functionally relevant proteins in hESC-RPE on different coatings after 8 weeks of culture. Scale bar 20 μm. Col—collagen IV, LN—laminin, Nid—nidogen-1.

We have previously shown that hESC-RPE express important RPE marker proteins post-cryopreservation when seeded on Col-IV + LN^32^. Before performing more detailed analyses on different coatings after cryopreservation, we compared the RPE characteristics of the cells on Col-IV + LN by the expression of cellular retinaldehyde-binding protein (CRALBP, involved in visual cycle) and Na^+^/K^+^-ATPase (important for transepithelial ion transport) and transepithelial electrical resistance (TER) before and after cryopreservation and found no major differences (Supplementary Fig. S1). In order to examine the development of barrier properties and integrity of the hESC-RPE monolayer on different coatings, TER was measured during the culture (Fig. 2a, Table 1). In concordance with the morphology of the epithelium, culturing cells on Col-IV alone resulted in low TER reaching a maximum (mean ± SEM) of 56 ± 7 Ωcm^2^ at 9 weeks. Surprisingly, compared to hESC-RPE seeded on Col-IV + LN and Col-IV + LN + Nid-1, TER remained lower on LN alone, reaching a maximum of 488 ± 27 Ωcm^2^ at 7 weeks after which TER values began to decline. Combining Col-IV and LN yielded epithelia with TER reaching 617 ± 18 Ωcm^2^ by week 7 after which TER plateaued. The highest TER values (731 ± 19 Ωcm^2^ at 9 weeks) were observed with hESC-RPE seeded on Col-IV + LN + Nid-1 coating.

Because Col-IV coating alone constantly failed to support formation of an intact hESC-RPE monolayer with proper morphology, this coating condition was excluded from the subsequent analyses. The level of hESC-RPE maturation on different coatings (Table 1) was further evaluated by expression and subcellular localisation of marker proteins important for RPE functionality. Due to the positive effect of the ECM linker protein Nid-1^33^ for barrier formation observed with TER measurements, the subsequent experiments were performed with an additional 10-times higher concentration of Nid-1 (Col-IV + LN + 10xNid-1) to examine if Nid-1 concentration affected RPE maturation and functionality. After 8 weeks in culture, CRALBP, Na^+^/K^+^-ATPase and tight junction proteins claudin-3, claudin-19 (Fig. 2b), as well as tight junction associated protein zonula occludens-1 (ZO-1, Supplementary Fig. S2), were expressed on all coatings. Interestingly, the junctional localisation of claudin-19 was consistently less frequent on LN alone compared to other coatings and improved by the addition of Nid-1 (both 1x  and 10x ), being in line with the TER measurements. Overall, based on TER measurements and immunostainings, adding complexity to the protein coating enhanced or at least accelerated the maturation of hESC-RPE barrier properties.

### Functionality of the hESC-RPE is affected by the culture substrate coating

**Figure 3:**
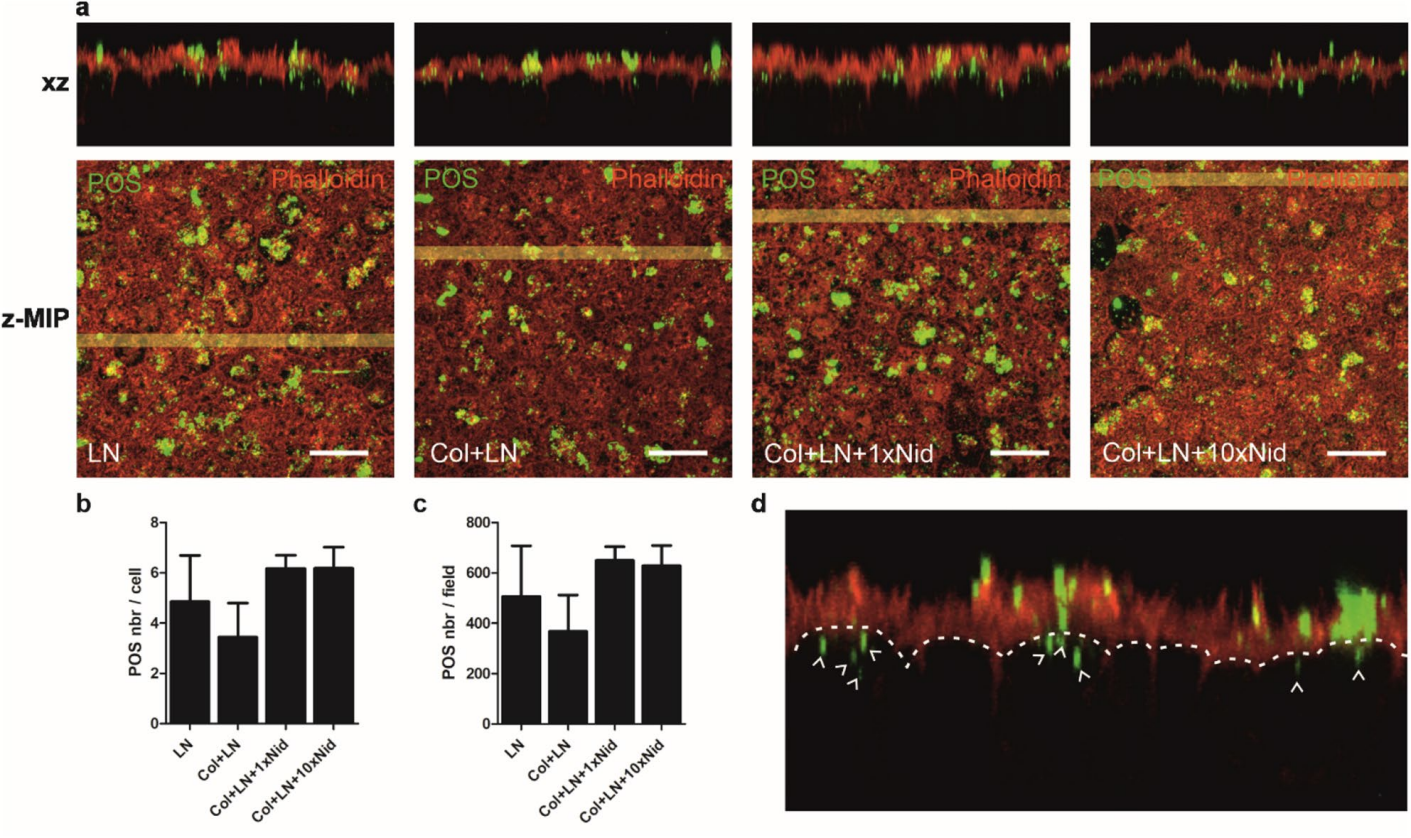
POS phagocytosis assay performed on hESC-RPE 13 weeks post-seeding with purified porcine POS (hESC-08/017, Table 1). (**a**) Representative laser scanning confocal microscopy z-maximum intensity y projections (z-MIP) and xz cross-sections (MIP from 20 sections) after 2-h phagocytic challenge showing the overall number, distribution and internalisation of the POS particles. The POS particles were labelled with anti-opsin (green) and filamentous actin with phalloidin (red). Scale bar 20 μm. Quantification of internalised POS particles (**b**) per cell and (**c**) per field. Each field contains cs. 100 cells. Internalised POS particles were manually calculated from each xz-MIP image (from five z-MIP images/coating). Both data represent means ± SD. The number of POS particles per cell between LN + Col and LN + Col + 1xNid (p = 0.0079) and LN + Col + 10xNid (p = 0.0079) was statistically significant. Similarly, POS number per field on Col + LN was statistically significant compared to LN + Col + 1xNid (p = 0.0079) and LN + Col + 10xNid (p = 0.0159). Statistical analysis was performed with Mann–Whitney U test. **(d)** A schematic image to demonstrate which POS particles (green) were calculated as internalised POS particles (white arrows). Col—collagen IV, LN—laminin, Nid—nidogen-1.

Daily renewal of POS and the subsequent removal of the shed POS by RPE via phagocytosis is essential for vision, phagocytosis thus being one of the key indicators for functional RPE. The phagocytic capacity of the hESC-RPE (Table 1) on different coatings was studied with isolated porcine POS that were incubated with the cells for 2 h. This was followed by labelling the POS with anti-opsin (Fig. 3a) and counting the number of internalised particles (Fig. 3b-d). POS phagocytosis was observed in hESC-RPE on all culture substrate coatings with slight differences between the coatings. The mean number of internalised particles was lowest on Col-IV + LN and highest on Col-IV + LN + Nid-1 (both 1x and 10x) while the variation was highest on LN and lowest on Col-IV + LN + Nid-1 (both 1x  and 10x).

**Figure 4:**
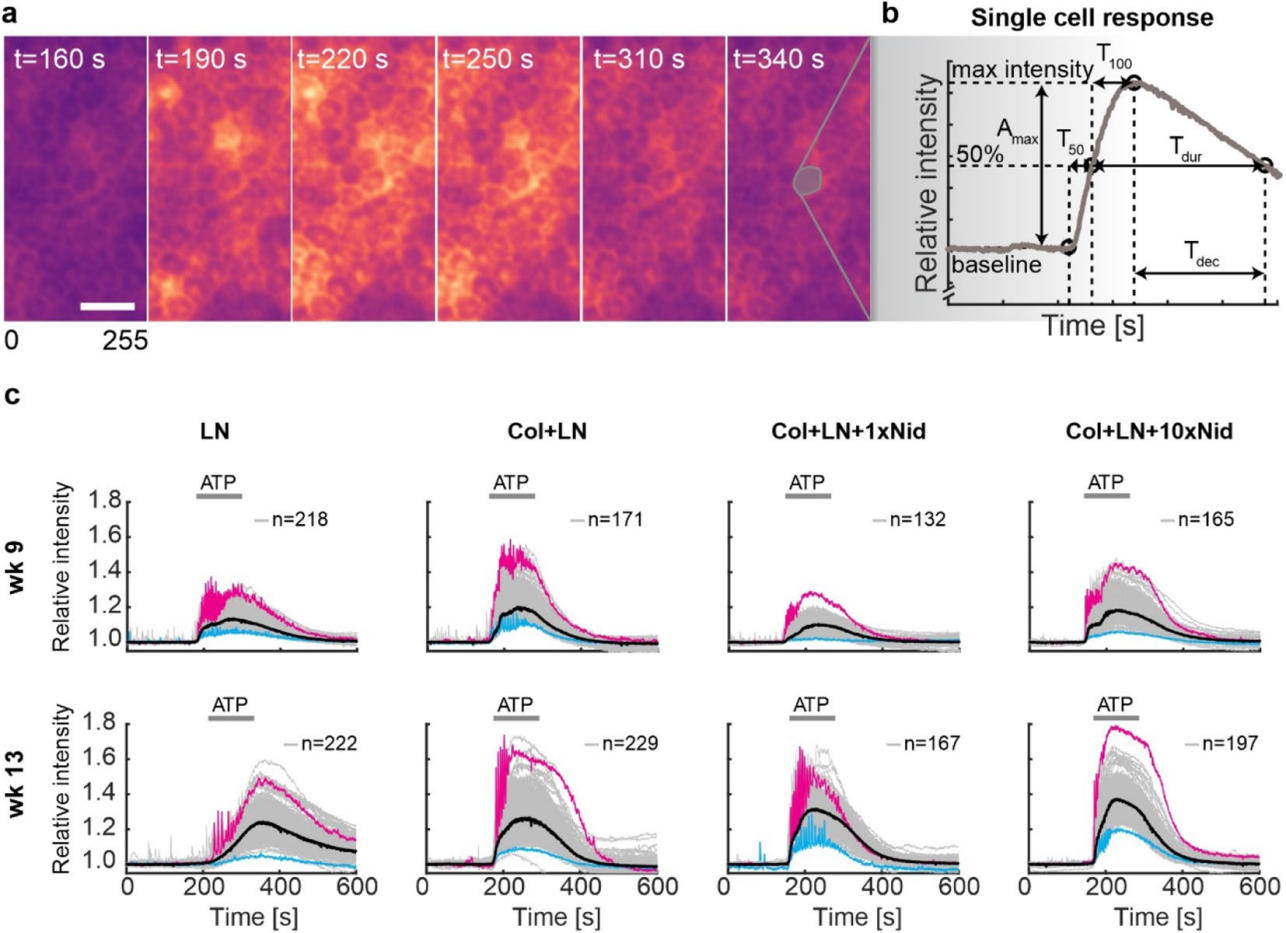
Ca^2+^-imaging response characterization and grouping. (**a**) Pseudocolored image time series of ATP-induced Ca^2+^ response in hESC-RPE with Fluo-4 Ca^2+^-indicator from the LN + Col + 10xNid surface. The pseudocolored intensities are linearly scaled from 0 to 255. Scale bar 10 µm. (**b**) Schematic curve representing the parameters calculated from a single cell Ca^2+^-response: maximum relative intensity (A_max_), first (T_50_) and second (T_100_) half of intensity rise time, intensity decay time from maximum to 50% (T_dec_) and response duration at 50% intensity (T_dur_). (**c**) The largest response groups with relative intensity as a function of time for each surface at 9 and 13 weeks. The parameters from (b) were used as inputs in the grouping algorithm. From each group, the strongest (magenta) and weakest (blue) responses are highlighted, in addition to the calculated median curve of the population (black). Col—collagen IV, LN—laminin, Nid—nidogen-1.

**Figure 5:**
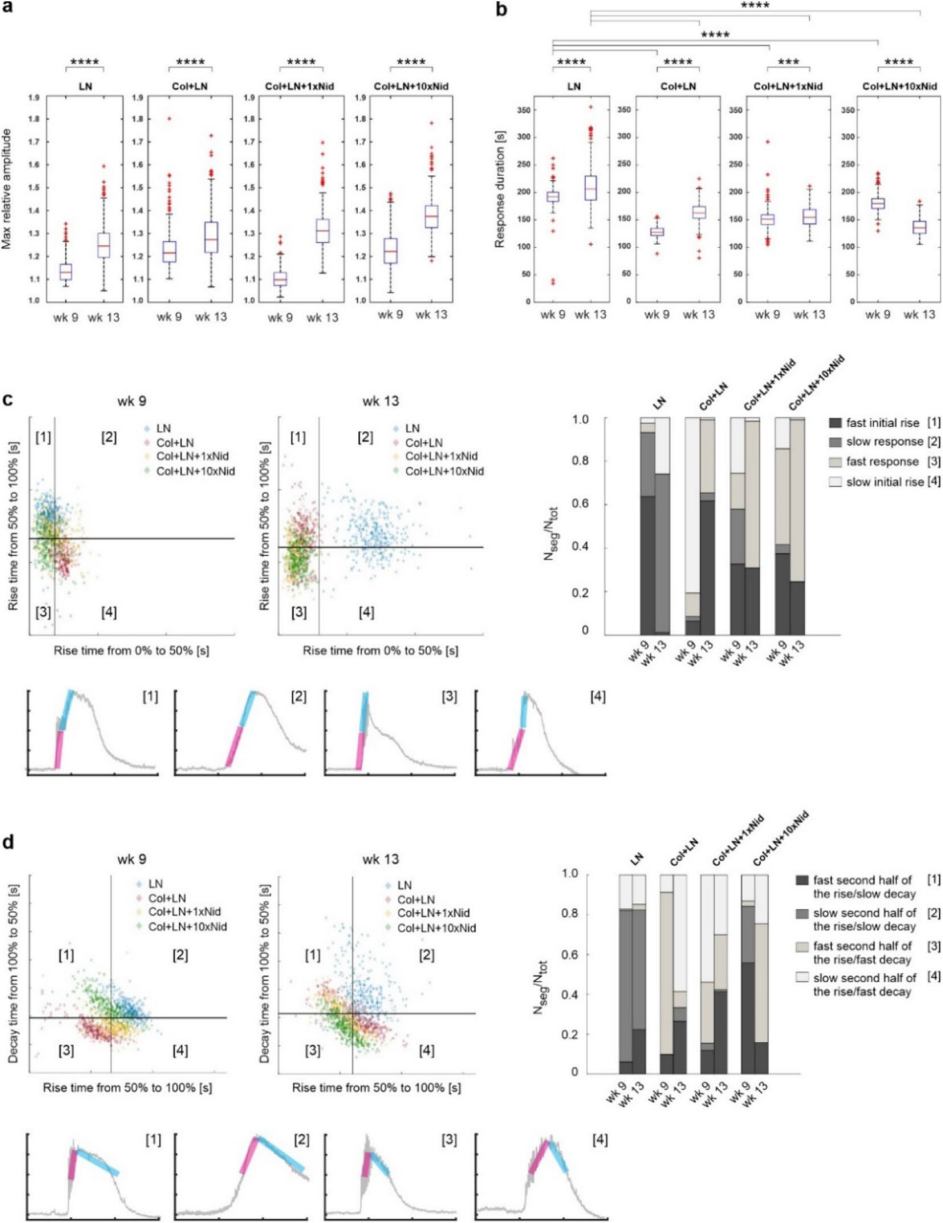
Visualization of the Ca^2+^ response parameters. Boxplots of maximum relative amplitude (**a**) and response duration at 50% intensity (**b**) at weeks 9 and 13. The red line represents the median value and the box extends to 25th and 75th percentiles. The red crosses mark values considered as outliers, outside approximately 99% of the population. (**c**) Upper left: Scatter graph between the first half (horizontal axis) and the second half (vertical axis) of the response rise time at weeks 9 (left) and 13 (right). The scatter fields are divided into four segments by taking the average value of each axis (black lines). Upper right: The number of responses in each segment per total number of responses is represented as a stacked bar graph for each culture surface coating at weeks 9 and 13 (right). Lower left: Examples of responses characterizing each segment. The response segment for the horizontal axis parameter is highlighted with a magenta bar and for the vertical axis parameter with a cyan bar. (**d**) Similar scatter and stacked bar graphs with example responses as in (c) for the second half of the response rise time (horizontal axis) and the intensity decay time from maximum to 50% intensity (vertical axis). ***p < 0.001, ****p < 0.0001 (Mann–Whitney U). Col—collagen IV, LN—laminin, Nid—nidogen-1.

Ca^2+^ acts as an important second messenger in diverse signalling pathways and the control of intracellular free Ca^2+^ concentration ([Ca^2+^]_i_) is involved in regulation of the majority of cellular reactions, including many RPE functions vital for the maintenance of healthy retina^34,35^. In our study, the RPE cells were loaded with the cell permeable Fluo-4 AM Ca^2+^ indicator dye, and with fluorescence time-lapse microscopy, we were able to monitor relative changes in [Ca^2+^]_i_ to analyse hESC-RPE Ca^2+^ dynamics on different protein coatings (Fig. 4a). We have previously developed tools to analyse Ca^2+^ imaging data from large populations of individual cells in intact RPE monolayers (Fig. 4b)^29^. These tools were applied here to identify population level events and potential differences between the culture conditions. We focused on ATP-induced purinergic signalling pathway that reflects both Ca^2+^ release from intracellular stores and its influx from the extracellular solution^36^. The hESC-RPE cells (Table 1) were exposed to 100 µM ATP for 2 min and several aspects of the recorded Ca^2+^ response were analysed including the amplitude as well as the rise and decay kinetics. Overall, on all surface coatings, an exposure to extracellular ATP induced Ca^2+^ responses that showed a wide cell-to-cell variation (Fig. 4c). Typical to RPE cells, most of the responses were biphasic with fast initial rise resulting from the release of Ca^2+^ from the intracellular stores followed by a slower secondary phase where extracellular Ca^2+^ influx plays a role. As the hESC-RPE cells matured in culture, the mean amplitude of the responses was observed to increase on all surfaces (Fig. 5a). However, on LN, the duration of the response was delayed compared to other surfaces, especially after long-term (13 weeks) culture (Figs. 4c, 5b). Further analysis of the response kinetics showed that purinergic Ca^2+^ signalling is altered on LN: although the majority of the hESC-RPE cells on this coating produced a fast initial [Ca^2+^]_i_ rise during early phases of maturation, after long-term culture, only few fast responding hESC-RPE cells with intact response characteristics were detected (Fig. 5c). In addition, a longer culture time was accompanied by the lengthening of the decay phase in the majority of cells cultured on LN (Fig. 5d) as well as by the more heterogenous response properties visible in the scatter graphs as more spread-out distribution (Fig. 5c, 5d). Addition of Col-IV to the coating together with LN supported the preservation of the fast initial response component better than LN alone during maturation. Although the number of cells with fast initial [Ca^2+^]_i_ rise on Col-IV + LN during early maturation (week 9) was reduced compared to LN alone, in the majority of cells at 13 weeks of culture, the fast response component was observed, unlike on LN alone (Fig. 5c). The response decay time on the Col-IV + LN surface coating was also shorter than on LN alone in both time points (Fig. 5b,d). Adding yet more complexity to the protein coating with Nid-1 (1x and 10x) increased the number of cells with the fast initial [Ca^2+^]_i_ rise and cell population homogeneity compared to Col-IV + LN (Fig. 5c). The higher Nid-1 concentration was beneficial for production of fast Ca^2+^ responses especially during the earlier phases (week 9) of RPE maturation (Figs. 4c, 5c), and this culture coating was superior in supporting cells with fast decay after long-term culture (week 13). Taken together, in addition to the barrier properties and phagocytosis, adding complexity to the protein coating also increased functional maintenance and cell–cell homogeneity of hESC-RPE in terms of Ca^2+^ signalling.

## Discussion

Although several clinical trials exploring the safety and surgical methods of hPSC-RPE transplantation have not revealed any major complications, the efficacy of RPE cell therapy remains to be evaluated^37^. For successful outcome, the transplanted cells need to be able to perform key RPE functions. Currently, several protocols for differentiation and culture of hPSC-RPE exist, utilising a variety of culture substrates and surface protein coatings, but lacking any comparative studies on how these culture conditions affect the functionality of hPSC-RPE. In this study, we examined the functional consequences of different cell culture surface protein coatings in the course of hESC-RPE culture and maturation, concentrating on the development of barrier properties, phagocytosis and ATP-induced Ca^2+^ response characteristics. In the Ca^2+^ signalling analysis, emphasis was put on analysing Ca^2+^ imaging data obtained from a large number of individual cells to obtain population level data. As cell banking with cryopreservation is an essential step in the production of cells for therapeutic applications, the study was conducted with cryopreserved cells. Previously, cryopreserved hESC- and human induced pluripotent stem cell (hiPSC)-derived RPE cells cultured on coatings like Matrigel and CellStart or more defined coatings like vitronectin or Col-IV + LN have been reported to maintain key RPE characteristics such as expression of functionally important proteins, phagocytosis and to some extent growth factor secretion^32,38–40^. However, to the best of our knowledge, there are no comparative studies on the functional consequences of different protein coatings after cryopreservation.

Since cell adhesion initiates a myriad of signalling cascades determining cell functions such as survival, growth or differentiation, we first compared the attachment of hESC-RPE on different basement membrane components. Cell attachment is mediated by multifunctional, heterodimeric transmembrane receptors, integrins. Previous studies of integrin expression in human RPE have identified several subunits which are highly expressed and bind to ECM proteins in Bruch’s membrane^10,11,16,41^. For this current study, we included Col-IV, LN and Nid-1. LN type LN521 was chosen because it is one of the main isoforms in Bruch’s membrane and has been previously shown to support differentiation and maturation of hESC-RPE^10,42^. When Col-IV coating alone was used, cell density post-thawing was low and spreading poor, unlike on other coatings. Despite similar adhesion strength among coatings, formation of an intact epithelium was hindered on Col-IV alone. This was somewhat surprising, since previously, the culture of freshly differentiated hESC-RPE on Col-IV has been successful^20^ and cultured RPE cells typically express several integrins binding both to collagens (α2β1) and laminins (α3β1, α6β1)^10,11^. LN on the other hand supported cell attachment and formation of an intact epithelium. Preference for binding LN instead of Col-IV has also been reported for a RPE cell line ARPE-19 by Aisenbrey et al^10^. The dominant role of LN in epithelial cell adhesion and formation of a functional basement membrane^43,44^, may partly explain why LN as the coating matrix improves cell attachment dramatically. The hESC-RPE cells on LN were also more pigmented than on other coatings. The melanin pigment is an important factor in the RPE cells, minimizing light scatter and protecting the cells from cytotoxicity caused by light and inflammation^45^. In general, pigmentation has been considered as a differentiation marker for hPSC-RPE. However, the level of pigmentation in the in vitro hPSC-RPE cultures varies and the functional consequences of this remain elusive. It has been shown that increase in hESC-RPE pigmentation over time does not lead to significant changes in gene expression, suggesting that pigmentation does not reflect the maturation state of the cells^46^, at least not at transcriptome level. Interestingly, among the few genes that were significantly differentially expressed between lightly and highly pigmented cells were genes involved in Ca^2+^ signaling and adherens junction remodeling^46^.

Development of mature tight junctions is a crucial property of the RPE cells and required for maintenance of the outer blood-retinal barrier. Thus, the formation of barrier properties during maturation of hESC-RPE seeded on different culture surface coatings was followed. During development, the composition and functional properties of RPE tight junctions mature as the neural retina and choroid differentiate^47^. A gradual maturation of the tight junctions indicated by increase in TER over time can also be seen in the hESC-RPE cells in our culture system. Morphologically, on all other coatings except Col-IV alone, hESC-RPE formed an intact pigmented monolayer of cuboidal cells. However, TER of hESC-RPE seeded on LN alone was consistently lower compared to cells cultured on a combination of Col-IV and LN. In our previous study comparing the effects of ECM coatings using freshly differentiated cells cultured for 6 weeks, the lowest TER values were also obtained from cells seeded on human LN compared to other coatings, including Col-IV^20^. In the present study, the TER values on LN alone were not only lower than on other coatings but also started to decline after 7 weeks in culture. This phenomenon was not observed in the study by Hazim et al.^48^ with freshly differentiated hiPSC-RPE and mouse LN during 10-week culture. However, in their study, the hiPSC-RPE were cultured in the presence of serum. As serum contains ECM proteins and growth factors which LN is known to bind with high affinity^49^, this could explain why the TER values were maintained in serum culture but not in the more defined serum replacement based culture system used in our study. The TER values on LN521 used in our study during the first 4 weeks of culture were in line with a previous study by Plaza Reyes et al., also using serum-free culture conditions and LN521 for culture of hESC-RPE. However, in their work, the TER values were not reported after 4 weeks time point^42^. In contrast to Col-IV or LN alone, bringing more complexity and/or potentially increasing the amount of adsorpted protein in the coating by combining Col-IV and LN, led to higher TER that was maintained throughout the follow-up time of 9 weeks. In addition to TER, the barrier properties were evaluated by comparing the expression of the major human RPE claudins, claudin-3 and -19^21^, by hESC-RPE seeded on different coatings. Claudins are a family of transmembrane proteins that bring specificity to tight junctions in a highly sophisticated manner. Claudins are tissue- and developmental stage-specific with only a subset of the so far identified 27 mammalian claudins being expressed by RPE. Based on their microenvironment or differing extracellular regions, claudins can form several types of paracellular barriers and channels, making the junctions for example cation- or anion-selective^50,51^. In our culture system, the expression of claudins typically appears after 7–8-weeks maturation time, initially in patches of claudin-immunopositive cells and as the cells mature, spreading across the whole epithelium and localising to the tight junctions. In this present study, after 8-week maturation time, claudins were expressed by cells on all coatings but especially claudin-19 was localised at this time point to tight junctions more in cells seeded in the presence of Nid-1.

Further functional analysis by phagocytosis assay suggested improved functionality in cells cultured on more complex protein coatings containing 1x or 10x Nid-1 in terms of the number of internalised POS particles and increased cell population homogeneity. In addition to phagocytosis, functionality of especially the ATP-mediated purinergic pathway has been considered an important criterion when assessing the quality of hiPSC-RPE for clinical applications due to the important role of ATP in the regulation of RPE physiology ^5,26,52^. More generally, Ca^2+^ signalling is an important regulator of numerous cell functions, controlled by the temporal and spatial distribution of [Ca^2+^]_i_^53^. Yet, no comparisons have been reported of how different culture conditions such as culture surface coating affect hPSC-RPE Ca^2+^ signalling. We have previously reported marked cell–cell heterogeneity in ATP-induced Ca^2+^ responses regarding magnitude and response kinetics^29^. With the analysis tools developed^29^, we could parametrise the response heterogeneity which allowed us to further group the cells according to their response characteristics and to assess population data on a single cell level from the RPE monolayer. When following the hESC-RPE cultures in time, maturation of the cultures was accompanied by increased Ca^2+^ response amplitudes on all coatings. Interestingly, although the functional analyses by phagocytosis assay did not indicate major defects in phagocytosis by cells on LN, further Ca^2+^ signalling analysis revealed differences in the [Ca^2+^]_i_ increase and decay characteristics. Initially, despite poor barrier properties at the 8–9 week time point, majority of cells on LN alone produced fast initial [Ca^2+^]_i_ rise reflecting intact ATP-induced release of Ca^2+^ from the intracellular stores. However, further maturation on LN was followed by deceleration of the responses. This was evident both in the rise and decay phase of the responses, yet the decay was affected already in the early maturation stage (week 9) cultures. The ATP-induced Ca^2+^ response in RPE is a result of complex molecular cascade where ATP first binds to P2Y_2_ receptors resulting in IP_3_ increase, release of Ca^2+^ from the ER and subsequent activation of several transporters and ion channels. Delayed or altered kinetics of the response indicate impaired downstream signalling following the ATP binding, and in the eye, it would result e.g. in compromised fluid regulation of the subretinal space^25,26^. Ca^2+^ imaging also suggested increased cell population homogeneity with more complex protein coatings compared to LN alone in the cultures matured for 13 weeks. The TER and immunostaining studies on barrier properties suggested that adding Nid-1 to the coating both accelerated hESC-RPE maturation and supported the maintenance of the epithelium the best. These observations were further supported by the improved phagocytosis on Nid-1 and Ca^2+^ imaging data showing that compared to other coatings, on Nid-1, especially with the higher tested concentration, a larger number of cells produced fast responses during the earlier phases (week 9) of RPE maturation and also had a fast decay after long-term culture (week 13). Furthermore, our results indicate that analysing the population measurements on a single cell level is advisable as population-averaged measurements can mask cell–cell heterogeneity.

Acquisition of mature tight junctions and appropriate ATP-activated Ca^2+^ signalling in RPE are key for the correct formation of gradients that drive directional fluid transport between the neural retina and the choroid, which together with phagocytosis are essential for the maintenance of photoreceptors. Considering the central role of ECM and basement membrane interactions in cellular differentiation and signal transduction, it is not surprising that a more complex and thus native-like culture surface (Col-IV + LN + Nid-1) was beneficial for the development and maintenance of these characteristics by hESC-RPE in vitro.

## Methods

For all experiments, the used cell lines and number of replicates have been indicated in Table 1.

### Human ESC-RPE differentiation and culture

Derivation and characterization^54^ as well as culture, subsequent differentiation^55,56^ into hESC-RPE and cryopreservation^32^ of cell lines Regea08/017 (46,XX), Regea11/013 (46,XY), and Regea13/012 (46,XY), was carried out as previously described. For experiments, hESC-RPE were conventionally thawed and seeded as described under each analysis. Culture of hESC-RPE was performed in serum-free medium (KO-DMEM) consisting of KnockOut Dulbecco’s modified Eagle’s medium (DMEM) supplemented with 15% KnockOut serum replacement, 2 mM GlutaMAX, 0.1 mM 2-mercaptoethanol, 1% MEM non-essential amino acids, and 50 U/ml penicillin–streptomycin (all from Gibco, Thermo Fisher Scientific).

### Cell adhesion—adhesion force studies

For cell adhesion force tests, hESC-RPE (hESC-08/017, Table 1) were seeded at 150 000 cells/cm^2^ on Thermanox plastic coverslips (Thermo Fisher Scientific) dip-coated at + 4 °C overnight with Col-IV (Sigma Aldrich C5533, 10 µg/cm^2^) or LN521 (Biolamina, 0.75 µg/cm^2^) alone or in combination, with and without nidogen-1 (R&D Systems, 2570-ND, 2.5 µg/cm^2^). Cells were cultured in the KO-DMEM medium described above and analysis was performed 24 h after seeding. Justification of the method and formulas for calculating the cell adhesion force are described in^57^. Adhesion force was measured using a centrifugation system (Hettich Universal 320) as described before^57^ with centrifugation force 750 g for 5 min. Cell number of adhered cells before and after centrifugation was evaluated with LDH assay according to the online protocol of OPS diagnostics. The absorbance was detected at 492 nm wavelength using a microplate reader (Tecan Infinite M200Pro and Tecan i-control software). LDH activity was measured at three different time points: after 5 min, 10 min and 15 min of incubation. The measured LDH activity was correlated with the cell number from a cell standard curve prepared under the same conditions.

### Cell adhesion—immunolabeling

For immunolabeling vinculin and F-actin, hESC-RPE (hESC-08/017, Table 1) were seeded at 30 000 cells/cm^2^ on dip coated Thermanox plastic coverslips and cultured in the KO-DMEM medium as described above. Immunolabeling of cells was performed 24 h after seeding as previously described^58^ with the exception of not mounting the samples. Instead, the imaging was performed in PBS immediately after labelling. Antibody information and dilutions can be found in Table 2. Samples were incubated with primary antibodies at + 4 °C overnight, and with secondary antibodies for 1 h at RT. Nuclear label (Hoechst 33,342, 1:1000) and Phalloidin were stained simultaneously with the secondary antibody incubation. Images were captured with a confocal microscope (NikonEclipse TE2000-E, Nikon; 60 × oil immersion objective).

**Table 2:** Antibodies used in the study.

Antibody/dye name	Manufacturer	Host/clonality	Cat#	Primary/secondary	Dilution/ab amount
Vinculin	Sigma Aldrich	Rabbit polyclonal	V4139	Primary	1:400
CRALBP	Abcam	Mouse monoclonal	ab15051	Primary	1:500
Na + /K + -ATPase	Abcam	Mouse monoclonal	ab7671	Primary	1:200
Claudin-3	Invitrogen, Thermo Fisher Scientific	Rabbit polyclonal	34-1700	Primary	1:100
Claudin-19	R&D Systems	Mouse monoclonal	MAB6970	Primary	1:100
ZO-1	Invitrogen, Thermo Fisher Scientific	Rabbit polyclonal	61-7300	Primary	1:200
Opsin	Sigma Aldrich	Mouse monoclonal	O4886	Primary	1:200
Phalloidin-Atto 550	Sigma Aldrich	–	19083	Primary	1:100
Anti-mouse IgG Alexa Fluor 488-conjugated	Molecular Probes, Thermo Fisher Scientific	Donkey polyclonal	A-21202	Secondary	1:400
Anti-rabbit IgG Alexa Fluor 488-conjugated	Molecular Probes, Thermo Fisher Scientific	Goat polyclonal	A-11034	Secondary	1:400
Anti-rabbit IgG Alexa Fluor 568-conjugated	Molecular Probes, Thermo Fisher Scientific	Goat polyclonal	A-11011	Secondary	1:400

### RPE functionality studies—hESC-RPE seeding and culture

For studying hESC-RPE functional properties, cells (Table 1) were seeded 200 000 cells/cm^2^ on 1 μm Millicell polyethylene terephthalate (PET) culture inserts (Millipore) dip-coated at + 4 °C overnight with Col-IV (5 µg/cm^2^) or LN521 (1.8 µg/cm^2^) alone or in combination, with and without Nid-1 (2.5 µg/cm^2^). For phagocytosis and Ca^2+^ imaging studies an additional coating with Col-IV (5 µg/cm^2^) + LN521 (1.8 µg/cm^2^) + Nid-1 (25 µg/cm^2^) was used. The cells were matured in KO-DMEM culture medium described above for 8–13 weeks before end-point analyses. Medium was changed 3 times a week.

### RPE functionality studies—TER

The barrier function of hESC-RPE (hESC-08/017, hESC-11/013, hESC-13/012, Table 1) on PET was assessed by TER measurements with a Millicell electrical resistance volt-ohm meter (Merck Millipore). Each hESC-RPE sheet was measured at least twice and the average TER values (Ωcm^2^) were calculated by subtracting the background TER (PET without cells) and multiplying the result by the surface area of the substrate. Data was measured at five time points (Table 1).

### RPE functionality studies—immunolabeling

Immunolabeling of hESC-RPE (hESC-08/017, hESC-11/013, Table 1) was performed as previously described^58^. Antibody information and dilutions can be found in Table 2. Samples were incubated with primary antibodies at + 4 °C overnight, and with secondary antibodies for 1 h at RT. Z-stack images were captured with a confocal microscope (LSM 800, Carl Zeiss; 63 × oil immersion objective).

### RPE functionality studies—phagocytosis assay

The porcine POS particles were isolated and purified as previously described^32^. The POS particles were fed to the hESC-RPE cells (hESC-08/017, Table 1) in the KO-DMEM medium supplemented with 10% foetal bovine serum and incubated for 2 h at + 37 °C in 5% CO_2_. Labelling with anti-opsin antibody (no POS primary antibody control in Supplementary Fig. S3) and Phalloidin (Table 2) was performed as preciously described^32^. Z-stack images were acquired with confocal microscope (LSM 800, Carl Zeiss, 63 × oil immersion objective) to visualise POS. For quantification of the internalised POS particles, z-stack images (5/coating) were resliced to 512 xz-slices. Maximum intensity projections (MIP) were sequentially generated in 20 xz-slice intervals using ImageJ. Internalised POS particles (illustrated in Fig. 3d) were manually calculated from each resliced xz-MIP. In addition, the cells were calculated from each z-stack image in order to obtain the average number of POS particles per cell.

### RPE functionality studies—Ca^2+^ imaging and data analyses

Human ESC-RPE cell (hESC-08/017, hESC-11/013, Table 1) Ca^2+^ dynamics was assessed with the Ca^2+^-sensitive dye fluo-4-acetoxymethyl ester (fluo-4 AM; Molecular Probes, Thermo Fischer Scientific) as previously described^29^. Briefly, the samples were washed with Elliot solution (pH 7.4, 330 mOsm) followed by incubation in 1 mM fluo-4 AM in Elliott buffer for 45 min at RT protected from light. During imaging, hESC-RPE cells were perfused with Elliot solution alone or Elliot containing 100 µM ATP (Sigma-Aldrich) using a gravity-fed solution exchange system (AutoMate Scientific). Imaging was performed at RT with Nikon Eclipse FN1 upright fluorescence microscope using a 25 × water immersion objective (NA = 1,10). The images were acquired every 500 ms with binning of 2 × 2 with Nikon Nis Elements Imaging Software (version 5,02). Excitation and emission wavelength of 494/506 nm for Fluo-4 was used with exposure time of 80 ms. 2 min of baseline imaging was performed before the cells were exposed to ATP for 2 min followed by imaging for additional 6 min in Elliot.

For data analysis, three 200 × 200 pixel (104 × 104 µm) regions of interest (ROIs) were cropped from each Ca^2+^ image stack in ImageJ and 70–120 individual cells were outlined from each ROI. The average intensity values of each cell as a function of time were extracted, and the intensity data was analysed using a self-developed MATLAB script package^29^ (MATLAB R2017b, The MathWorks Inc.). For each cell response, a set of quantities to describe the intensity amplitude and dynamics was calculated: maximum relative amplitude, first (0% to 50% intensity) and second half (50% to 100%) of the intensity rise time, time to maximum amplitude (0% to 100%), decay time (100% to 50%) and response duration at 50% intensity (Fig. 4b). After the single-cell analysis, the intensity responses were sorted with a clustering algorithm, using the calculated intensity parameters of all cells from the three ROIs as input. As a result, the algorithm provided two to four groups of intensity responses having a unique set of response characteristics. The largest group of each measurement was chosen to represent the behaviour of each cell population (Fig. 4c).

### Image processing

Images were processed with ImageJ^59,60^ using only linear brightness and contrast adjustments for the pixel intensities. Final figures were generated using GraphPad Prism version 8 for Windows (GraphPad Software, La Jolla, CA, USA) and CorelDRAW Graphics Suite 2019 (Corel corporation, Ottawa, Canada).

### Statistical analysis

Normality was tested with Shapiro–Wilk test and following statistical analysis between two groups was performed with the unpaired Mann–Whitney U test using GraphPad Prism. A p value of < 0.05 was considered statistically significant.

### Ethical issues

Tampere University has the approval of the National Supervisory Authority for Welfare and Health Valvira (Dnro 1426/32/300/05) to conduct research on human embryos. The institute also has supportive statements of the Ethical Committee of the Pirkanmaa Hospital District to derive, culture, and differentiate hESC lines (Skottman/R05116). No new cell lines were derived for this study.

## Supplementary information

Supplementary information.

## Data Availability

The datasets generated during and/or analysed during the current study are available from the corresponding author on request.
