# Supplementary material for: Culture surface protein coatings affect the barrier properties and calcium signalling of hESC-RPE
Source: Sci Rep. 2021 Jan 13;11:933. doi: 10.1038/s41598-020-79638-8 (PMC7806758; doi:10.1038/s41598-020-79638-8)
Supplement: Supplementary file 1 — Supplementary information. [file 41598_2020_79638_MOESM1_ESM.pdf]

Supplementary information

Culture surface protein coatings affect the barrier properties and calcium signalling of hESC-RPE

Taina Viheriälä[1§], Juhana Sorvari[1§], Teemu O. Ihalainen[1], Anni Mörö[1], Pyry Grönroos[1], Sabrina Schlie-Wolter[2], Boris Chichkov[3], Heli Skottman[1], Soile Nymark[1#], Tanja Ilmarinen[1#*]

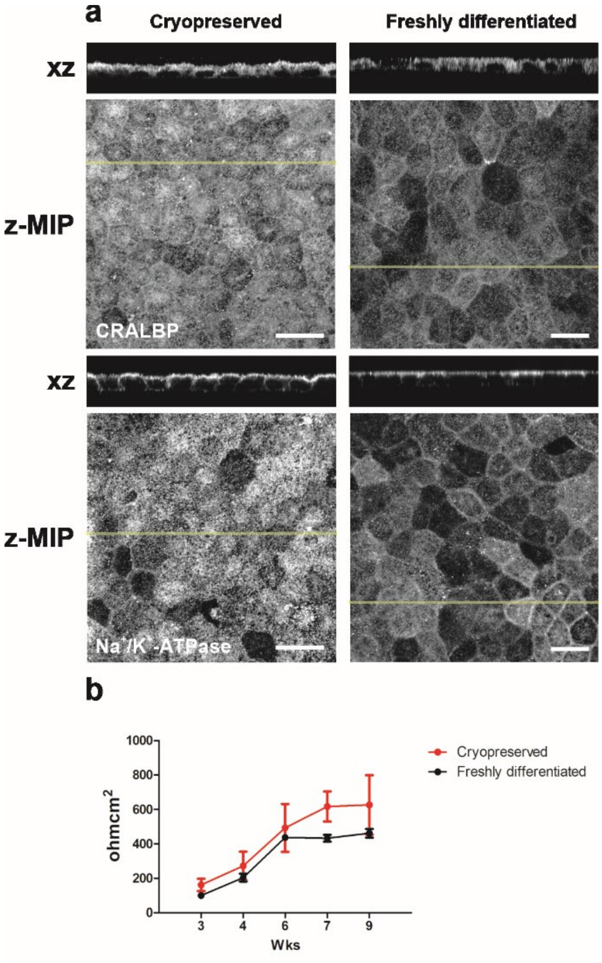

Figure S1. Barrier properties of cryopreserved and freshly differentiated hESC-RPE (hESC-08/017) on LN+Col at 8 weeks. (**a**) Representative laser scanning confocal microscopy z-maximum intensity projections (MIP) and xz cross-sections (MIP from 10 sections) showing expression and localisation of CRALBP and Na^+/K^+-ATPase. Scale bar is 20 µm. (**b**) TER of cryopreserved and freshly differentiated hESC-RPE on LN+Col. 5 technical replicates were used at all time points in freshly differentiated cells. In cryopreserved cells, 3 hESC-RPE lines from 6 biological and 24 technical replicates (as in Figure 2). Data represents means ±SD at 5 time points.

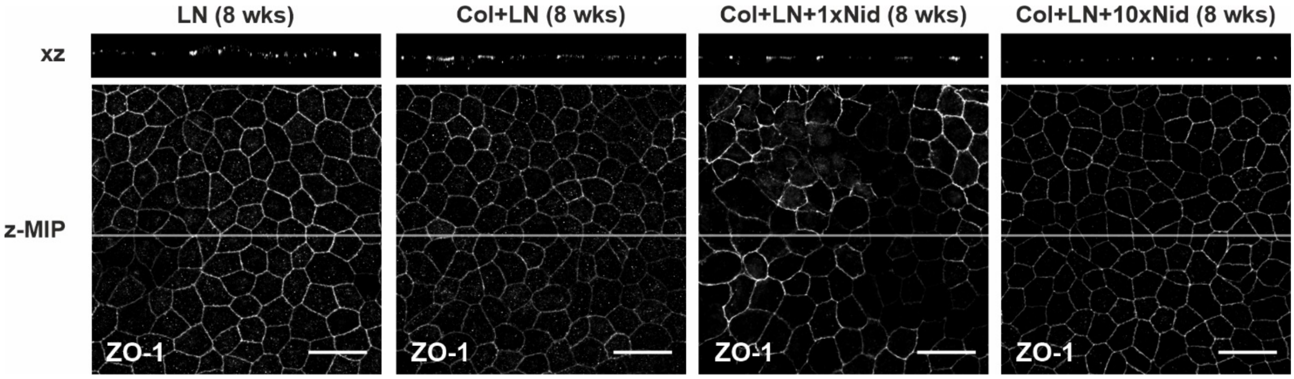

Figure S2. Barrier properties of hESC-RPE on different coatings. Representative (shown for hESC-08/017, Table 1) laser scanning confocal microscopy z-maximum intensity projections (MIP) and xz cross-sections (MIP from 10 sections) showing expression and subcellular localisation of ZO-1 in hESC-RPE on different coatings after 8 weeks of culture. Scale bar 20 µm. Col - collagen IV, LN - laminin, Nid - nidogen-1.

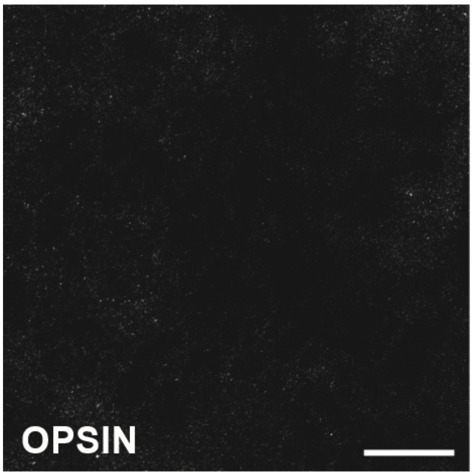

Figure S3. Control staining of opsin. hESC-RPE (hESC-08/017) without POS feeding immunolabelled with opsin antibody and imaged with fluorescence microscope (LSM800, 63x oil immersion objective). Scale bar 20 µm.
